# Sex differences in the association between asthma incidence and modifiable risk factors in Korean middle-aged and older adults: NHIS-HEALS 10-year cohort

**DOI:** 10.1186/s12890-019-1023-3

**Published:** 2019-12-16

**Authors:** Susan Park, Sun-Young Jung, Jin-Won Kwon

**Affiliations:** 1 College of Pharmacy and Research Institute of Pharmaceutical Sciences, Kyungpook National University, 80 Daehak-ro, Daegu, 41566 South Korea; 2 College of Pharmacy, Chung-Ang University, Seoul, Republic of Korea

**Keywords:** Body mass index, Waist circumference, Asthma, Older adults

## Abstract

**Background:**

This study investigated the sex-specific incidence of asthma and the effects of modifiable risk factors, particularly obesity, on asthma incidence among middle-aged and older individuals in Korea.

**Methods:**

We used data from the National Health Insurance Service-Health Screening Cohort (NHIS-HEALS), which includes health examinees aged 40–79 years in 2002–2003. In total, 459,529 participants with baseline anthropometric measurements were followed-up for 10 years and the development of asthma was evaluated (2004–2013). For subgroup analysis, 246,019 participants who had body mass index (BMI) and waist circumference (WC) measurements taken in 2008–2009 were included in the analysis of the asthma incidence for 2010–2013. Factors associated with asthma were analysed using Cox proportional hazard models.

**Results:**

The cohort comprised 4,248,813 (men, 2,358,541; women, 1,890,272) person-years of follow-up for 2004–2013. The asthma incidence was 10.58 and 15.03 per 1000 person-years for men and women, respectively. Asthma incidence increased with age, notably so in men. Obesity based on the baseline BMI was significantly associated with asthma development in both sexes (men, HR = 1.23, 95% confidence interval (CI) = 1.13–1.34; women, HR = 1.40 95% CI = 1.32–1.48). High WC was also related to asthma incidence in both sexes with statistical significance (men, HR = 1.34, 95% CI = 1.16–1.57; women, HR = 1.19 95% CI = 1.03–1.37). Analysis of the combined effects of BMI and WC showed that men had a higher asthma risk in the group with both general obesity and abdominal obesity than in the group with non-abdominal obesity and normal BMI. However, obese women had a higher risk of asthma regardless of abdominal obesity. Similarly, smoking was associated with asthma in both sexes but drinking and physical activity showed different associations between the sexes.

**Conclusions:**

Our results revealed that asthma incidence was substantially high at old age and lifestyle factors were associated with asthma development. Practical strategies including weight control and healthy lifestyle modification are required to prevent asthma in older people.

## Background

Asthma in older adults is a serious health problem, particularly in conjunction with the rapid aging of society [1]. The prevalence of asthma among older adults is substantially high (range, 4.5–12.7%) [2, 3], even though asthma is more likely to be underdiagnosed and undertreated [4]. Moreover, older patients with asthma are more likely to be hospitalised and have a higher death rate than their younger counterparts [5, 6]. Consequently, the burden of asthma increases with age in adult individuals and this is more apparent among elderly individuals [7, 8]. Nevertheless, basic epidemiological data on the prevention and treatment of asthma among older adults are limited [2, 9].

Lifestyle factors such as smoking, drinking and physical activity are reportedly modifiable risk factors for adult asthma. However, there are inconsistencies in their associations according to sex [10]. Two previous longitudinal studies reported that physical activity was not associated with asthma incidence among middle-aged and older women [11, 12], whereas a Finnish cohort study showed that physical activity had a protective effect on asthma onset in men [10]. Furthermore, previous studies have indicated the possibility of sex differences in the effects of smoking and drinking on asthma development [13, 14], but the available population-based studies are insufficient, particularly for older people.

Obesity is another well-known modifiable risk factor for asthma [15–27]; however, age- and sex-related differences in the obesity–asthma association have not been fully elucidated. Although the adverse effects of high body mass index (BMI) were more evident among elderly subjects than young and middle-aged participants in a recent study [15], data on the association between obesity and asthma in older adults remains sparse. In terms of sex, some cohort studies have shown sex-specific associations between high BMI and asthma development in women [16–21] and men [22]. However, others have reported a similar relationship between BMI and asthma for both sexes [23–27].

BMI is a commonly employed tool for measuring obesity in adults. However, it cannot reflect the distributions of lean mass and fat in body compartments, which are significantly influenced by age and sex [28, 29]. Alternatively, waist circumference (WC) is used as a stronger predictor of visceral adipose tissue (VAT), which is more metabolically active than fat at other sites [30, 31] compared to BMI [32, 33]. To this end, using both WC and BMI to measure obesity is recommended [34, 35]. However, few studies have taken advantage of both BMI and WC to assess the risk of asthma [36–39].

Therefore, this study aimed to assess the asthma incidence according to both age and sex while also investigating the sex-specific associations between modifiable risk factors and asthma incidence in Korean middle-aged and older adults. In particular, the association between obesity and asthma using individual and combined measurements of BMI and WC was explored.

## Methods

### Study participants

The National Health Insurance Service–Health Screening Cohort (NHIS-HEALS) is a cohort comprising a 10% random sample (*n* = 514,866) from all health-screened participants aged 40–79 years from 2002 to 2003 [40]. The NHIS provides biennial health screening programmes (annually for manual workers) for insured individuals and their dependents aged ≥40 years. This NHIS covers approximately 97% (Medicaid, approximately 3%) of Korea’s entire population and the participation rate among the eligible population in the NHIS health screening programme was 74.8% in 2014 [40]. The screening programme included anthropometric measurements, laboratory assessments of blood and urine samples, and the administration of questionnaires about health behaviours. The NHIS-HEALS contains data on health screening, insurance eligibility status, household income, demographic information, medical treatments and mortality information, such as the cause and date of death, for 2002–2013.

Except for participants without BMI measurements (*n* = 516), the number of participants who had their BMIs measured from 2002 to 2003 was 514,350. Among them, participants who had extreme BMI values (*n* = 17) and those who had a diagnosis of asthma (*n* = 53,779) or had died (*n* = 1025) in 2002–2003 were excluded. Finally, 459,529 participants in total were analysed as the baseline study sample.

**Fig. 1:**
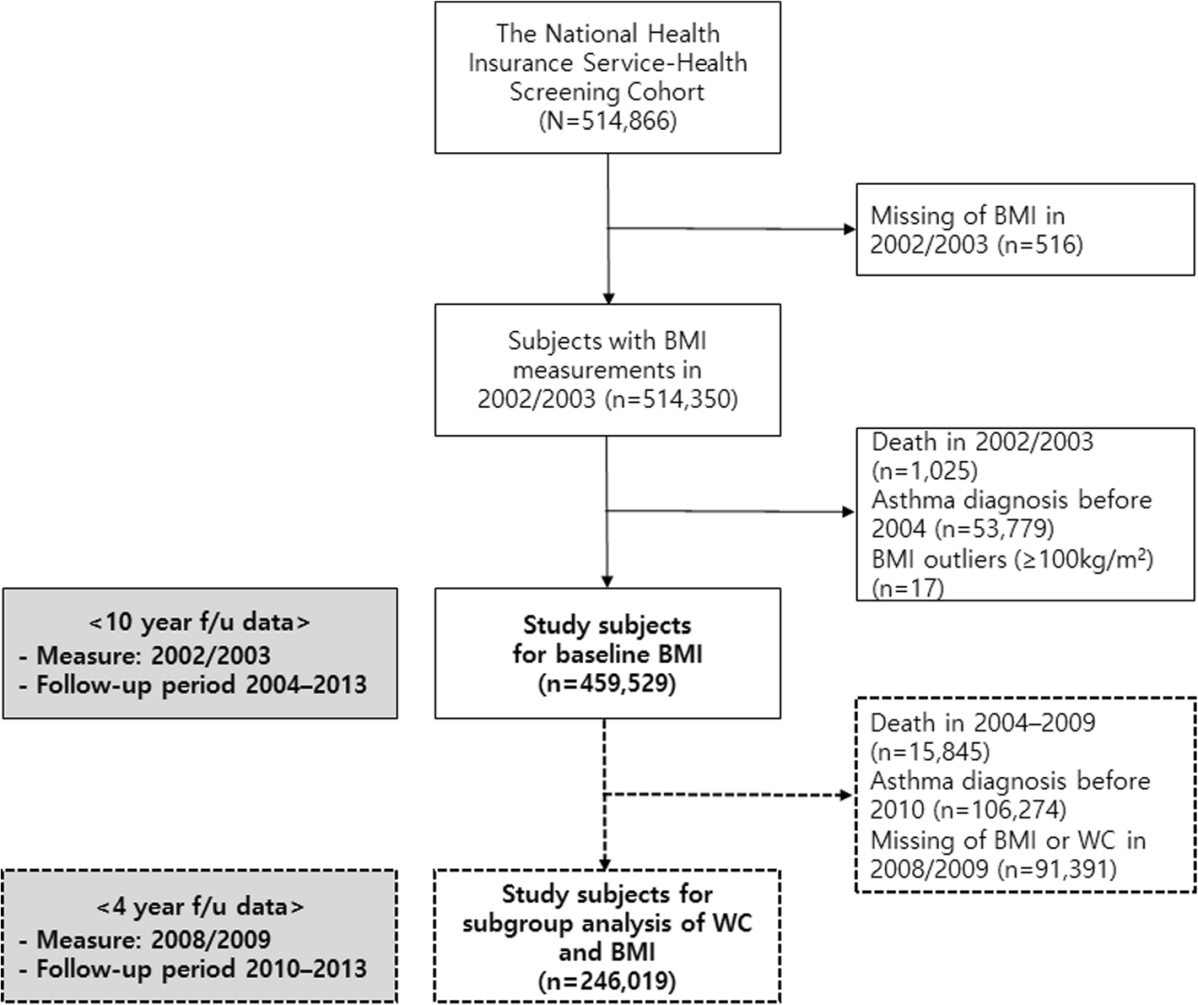
Flow diagram for the subjects included in this study

The measurement of WC was first included in NHIS-HEALS in 2008. Therefore, we selected 246,019 out of 459,529 participants for subgroup analysis to investigate the combined effects of BMI and WC on asthma after excluding participants with missing WC or BMI values in 2008–2009 (*n* = 91,391) and those who had an asthma diagnosis (*n* = 106,274) or had died (*n* = 15,845) before the follow-up period (Fig. 1).

In the sample selection process, we excluded patients with an asthma diagnosis regardless of drug prescriptions to rule out the possibility of pre-existing asthma before the follow-up period.

### Measures

A newly diagnosed asthma case was defined as someone who has both an asthma diagnosis and a prescription for asthma drugs to increase diagnosis accuracy [41]. Asthma diagnoses were assessed using the J45 or J46 ICD-10 codes (International Classification of Disease, 10th Edition) in medical treatment data. Asthma drugs included inhaled beta-agonists, inhaled corticosteroids and leukotriene receptor antagonists according to the Global Initiative for Asthma Guidelines.

Anthropometric indexes including height, weight and WC were measured in a standardised manner by trained nurses at each examination [34]. General obesity was determined from BMI, calculated as weight in kilograms divided by the square of height in meters, and the measurements were classified into five groups based on the obesity criteria for Asians: 1) < 18.5 kg/m^2^ (underweight), 2) 18.5 to < 23 kg/m^2^ (normal), 3) 23 to < 25 kg/m^2^ (overweight), 4) 25 to < 30 kg/m^2^ (obesity) and 5) ≥30 kg/m^2^ (severe obesity) [42]. The measured WC was used to assess abdominal obesity, which was defined as WC > 90 cm in men and > 85 cm in women [43]. The combined variable of BMI and WC was categorised into six groups: 1) no abdominal obesity & underweight/normal BMI, 2) no abdominal obesity & overweight BMI, 3) no abdominal obesity & obese BMI, 4) abdominal obesity & underweight/normal BMI, 5) abdominal obesity & overweight BMI and 6) abdominal obesity & obese BMI.

Household income was divided into three groups (low: grade 0–4, middle: grade 5–8 and high: grade 9–10), based on 11 levels of insurance fees in the NHIS insurance eligibility data. Information on health behaviours such as smoking, drinking and physical activity was collected via questionnaires. Smoking status was categorised as current smoker, past smoker and non-smoker using the question “Do you smoke cigarettes now?” with three levels of responses: 1) never smoked, 2) smoked in the past, but not now and 3) currently smoke. The questionnaires regarding drinking and physical activity were assessed based on the frequency of behaviours and consisted of five levels: 1) none, 2) 2–3 times per month, 3) 1–2 times per week, 4) 3–4 times per week and 5) almost every day. Using these levels, the variables were categorised into none, < 2 times per week and > 3 times per week. The insurance types were: self-employed insured, employed insured and medical aid beneficiary.

### Analysis

The development of asthma was monitored from 1 January 2004 to 31 December 2013 for the analysis of baseline BMI and health behaviours. For subgroup analysis on both BMI and WC, the follow-up for asthma incidence started on 1 January 2010 and ended on 31 December 2013. The participants who had medical records with an asthma diagnosis code (J45 or J46) before starting the follow-up (1 January 2004 for the main analysis and 1 January 2010 for subgroup analysis) were excluded in the analysis to confirm the pre-existence of asthma. Asthma incidence was the end-point for all analyses and participants who were dead or did not have an asthma diagnosis by 31 December 2013 were censored.

First, the sample size and proportion of each category were calculated to check the distribution of the baseline population. We analysed the asthma incidence rates according to sex and age groups, which were calculated by dividing the number of new cases of asthma by the summing person-years of observation in each category during the follow-up period. Uni- and multi-variable hazard ratios (HRs) and their 95% confidence intervals (CIs) were estimated using Cox proportional hazard models to evaluate the effects of baseline BMI on asthma incidence. In the subgroup analysis, HRs were estimated to explore the effects of BMI and WC both separately and in combination in 2008/2009 on the development of asthma. Multivariable models were adjusted for age, insurance type, household income, smoking, drinking and physical activity at the baseline. To reduce data loss, missing values for smoking, drinking and physical activity were included in the analysis as a separate category named ‘unknown’.

Sensitivity analysis with various follow-up times was conducted to evaluate the induction period for obesity-induced asthma. We compared the effects of the baseline characteristics on the asthma incidence using two and four years of induction time before the asthma follow-up.

All analyses of men and women were conducted separately to explore the association between BMI and asthma incidence in terms of sex. Statistical analyses were performed using SAS version 9.4 (SAS Institute Inc., Cary, NC, USA).

## Results

The cohort comprised 459,529 participants (men, 254,643; women, 204,886) with 9.2 years of mean follow-up time. The study period counted as 4,248,813 person-years (men, 2,358,541 person-years; women, 1,890,272 person-years). During the observation period, 24,959 men and 28,412 women developed asthma. The distributions of the characteristics at the baseline are presented according to sex in Table 1. The mean ages at the baseline were 51.5 and 53.1 years in men and women, respectively. A higher prevalence of current smoking (men, 40.6%; women, 2.6%) and alcohol consumption (≥3 times a week: men, 19.4%; women, 1.8%) was observed more in men than in women. Moreover, the proportions of frequent physical activity (≥3 times a week: men, 20.1%; women, 15.9%) and obesity (BMI ≥25 kg/m^2^: men, 35.5%; women, 33.6%) were higher in men than in women.

**Table 1:** Baseline characteristics of study participants by sex

	Men	Women
	n (%)	n (%)
Total	254,643	204,886
Age		
40–49	128,281 (50.4)	90,458 (44.2)
50–59	71,765 (28.2)	58,433 (28.5)
60–69	43,079 (16.9)	42,448 (20.7)
≥ 70	11,518 (4.5)	13,547 (6.6)
Household income		
Low	62,487 (24.5)	75,521 (36.9)
Medium	97,249 (38.2)	70,298 (34.3)
High	94,907 (37.3)	59,067 (28.8)
Insurance type		
Self-employed insured	79,514 (31.2)	94,455 (46.1)
Employed insured	174,968 (68.7)	110,128 (53.8)
Medical aid beneficiary	161 (0.1)	303 (0.2)
BMI		
Underweight ( < 18.5 kg/m^2^)	5548 (2.2)	4647 (2.3)
Normal (18.5–< 23 kg/m^2^)	86,195 (33.9)	77,837 (38.0)
Overweight (23–< 25 kg/m^2^)	72,614 (28.5)	53,585 (26.2)
Obesity (25–< 30 kg/m^2^)	85,063 (33.4)	61,638 (30.1)
Severe obesity (≥ 30 kg/m^2^)	5223 (2.1)	7179 (3.5)
Smoking status		
Unknown	11,751 (4.6)	7766 (3.8)
Never smoker	101,698 (39.9)	189,987 (92.7)
Past smoker	37,778 (14.8)	1825 (0.9)
Current smoker	103,416 (40.6)	5308 (2.6)
Alcohol consumption		
Unknown	3612 (1.4)	5007 (2.4)
None	85,986 (33.8)	164,513 (80.3)
< Twice a week	115,545 (45.4)	31,637 (15.4)
≥ Three times a week	49,500 (19.4)	3729 (1.8)
Physical activity		
Unknown	7844 (3.1)	6031 (2.9)
None	121,735 (47.8)	133,098 (65.0)
< Twice a week	73,988 (29.1)	33,089 (16.2)
≥ Three times a week	51,076 (20.1)	32,668 (15.9)

BMI = Body Mass Index

Baseline characteristics were measured in 2002/2003

**Fig. 2:**
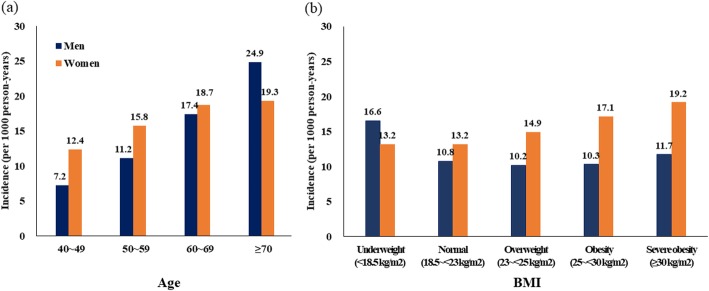
The asthma incidence per 1000 person-years during the 10-year follow-up period (2004–2013). (a) Asthma incidence by age and sex; (b) asthma incidence by BMI and sex.*An asthma case was defined as a person with an ICD-10 diagnosis code J45 or J46 and a prescription for inhaled beta-agonists, inhaled corticosteroid, or leukotriene receptor antagonist in the claims database

Figure 2 shows the incidence rates of asthma based on age and BMI by sex. The overall incidence rates were 10.6 and 15.0 per 1000 person-years in men and women, respectively. The asthma incidence rates were higher among women than among men for those aged 40–60 years. Although the incidence of asthma increased with age for both sexes, the increase was much steeper for men than for women. Therefore, the incidence rate was higher among men than women in individuals aged ≥70 years. In the BMI categories, the incidence of asthma was U-shaped in men. Underweight subjects had the highest incidence of asthma (16.6 per 1000 person-years) among the BMI ranges and the second-highest incidence was observed in the severe obesity group (11.7 per 1000 person-years). Meanwhile, the incidence of asthma increased monotonically with BMI in women and the highest incidence was observed in severely obese women (19.2 per 1000 person-years).

Table 2 presents the uni- and multi-variable HRs for the asthma incidence according to various risk factors based on sex. Severe obesity based on BMI was a common risk factor for asthma incidence in both sexes (BMI ≥30 kg/m^2^, men HR = 1.23, 95% CI: 1.13–1.34; women, HR = 1.40, 95% CI: 1.32–1.48). In women, BMI-based underweight status was associated with a lower risk of asthma compared to normal weight, whereas underweight men had an increased risk of asthma (men, HR = 1.32, 95% CI: 1.23–1.43; women, HR = 0.94, 95% CI: 0.87–1.03).

**Table 2:** Hazard ratios for asthma during a 10-year follow-up (2004–2013) according to age, economic status, health behaviours and BMI by sex

	Men			Women		
	Case	Univariable Model	MultivariableModel	Case	Univariable Model	MultivariableModel
		HR (95% CI)	HR (95% CI)		HR (95% CI)	HR (95% CI)
Age						
40–49	8924	–	–	10,602	–	–
50–59	7451	1.55 (1.51–1.60)	1.53 (1.48–1.58)	8547	1.28 (1.24–1.32)	1.24 (1.21–1.28)
60–69	6466	2.47 (2.39–2.55)	2.38 (2.30–2.46)	7119	1.53 (1.49–1.58)	1.48 (1.44–1.53)
≥ 70	2118	3.70 (3.53–3.88)	3.46 (3.30–3.64)	2144	1.61 (1.54–1.69)	1.58 (1.51–1.66)
Insurance type						
Self-employed insured	8927	–	–	13,309	–	–
Employed insured	16,000	0.78 (0.76–0.80)	0.92 (0.89–0.94)	15,045	0.96 (0.94–0.98)	1.00 (0.97–1.02)
Medical aid beneficiary	32	2.20 (1.56–3.12)	1.69 (1.20–2.40)	58	1.51 (1.17–1.95)	1.38 (1.06–1.78)
Household income						
Low	6913	–	–	10,520	–	–
Medium	9193	0.82 (0.80–0.85)	0.95 (0.92–0.98)	9756	0.99 (0.96–1.02)	0.99 (0.96–1.01)
High	8853	0.80 (0.77–0.82)	0.97 (0.94–1.00)	8136	0.98 (0.95–1.01)	1.00 (0.98–1.03)
BMI						
Underweight (< 18.5 kg/m^2^)	763	1.57 (1.46–1.69)	1.32 (1.23–1.43)	554	1.02 (0.94–1.10)	0.94 (0.87–1.03)
Normal (18.5–< 23 kg/m^2^)	8494	–	–	9571		–
Overweight (23–< 25 kg/m^2^)	6910	0.94 (0.91–0.97)	1.00 (0.96–1.03)	7390	1.14 (1.11–1.17)	1.11 (1.07–1.14)
Obesity (25–< 30 kg/m^2^)	8223	0.95 (0.93–0.98)	1.04 (1.01–1.07)	9657	1.31 (1.27–1.34)	1.24 (1.21–1.28)
Severe obesity (≥30 kg/m^2^)	569	1.08 (1.00–1.18)	1.23 (1.13–1.34)	1240	1.50 (1.42–1.58)	1.40 (1.32–1.48)
Smoking status						
Unknown	1158	0.95 (0.90–1.01)	1.05 (0.98–1.13)	1093	1.03 (0.97–1.09)	1.00 (0.92–1.10)
Never smoker	10,474	–	–	26,081	–	–
Past smoker	3803	0.97 (0.94–1.01)	1.11 (1.07–1.15)	269	1.09 (0.97–1.23)	1.10 (0.98–1.24)
Current smoker	9524	0.89 (0.87–0.92)	1.07 (1.04–1.10)	969	1.43 (1.34–1.53)	1.42 (1.33–1.51)
Drinking status						
Unknown	369	0.89 (0.80–0.99)	0.85 (0.76–0.96)	728	1.05 (0.97–1.13)	1.00 (0.90–1.12)
None	9749	–	–	22,950	–	–
< Twice a week	10,040	0.74 (0.71–0.76)	0.87 (0.84–0.89)	4180	0.93 (0.90–0.97)	0.99 (0.95–1.02)
≥ Three times a week	4801	0.85 (0.82–0.88)	0.86 (0.83–0.90)	554	1.07 (0.98–1.16)	1.05 (0.96–1.14)
Physical activity						
Unknown	798	0.97 (0.90–1.04)	1.07 (0.98–1.16)	866	1.04 (0.97–1.11)	1.04 (0.96–1.13)
None	12,572	–	–	18,364	–	–
< Twice a week	6518	0.82 (0.80–0.85)	0.96 (0.93–0.99)	4503	0.97 (0.94–1.01)	1.02 (0.99–1.06)
≥ Three times a week	5071	0.94 (0.91–0.97)	0.96 (0.93–0.99)	4679	1.03 (1.00–1.07)	1.05 (1.02–1.08)

BMI = Body Mass Index; HR = hazard ratios; CI = confidence intervals

Current smoking was a significant risk factor for asthma development in both sexes (men: HR = 1.07; 95% CI = 1.04–1.10; women: HR = 1.42; 95% CI = 1.33–1.51). Men who drank had a lower HR for asthma than those who did not (HR = 0.86; 95% CI = 0.83–0.90). However, a non-significant effect was observed in women. Physical activity had a preventive effect on the development of asthma in men (HR = 0.96; 95% CI = 0.93–0.99), whereas it was a risk factor for women (HR = 1.05; 95% CI = 1.02–1.08).

Table 3 presents the adjusted HRs for asthma development according to the individual and combined variables of BMI (general obesity) and WC (abdominal obesity). The HRs for obesity measured by BMI in 2008/2009 were similar to those for obesity measured by BMI in 2002/2003 in the main analysis (BMI ≥30 kg/m^2^, men HR = 1.23, 95% CI: 1.05–1.45; women, HR = 1.28, 95% CI: 1.12–1.47). High WC was significantly associated with asthma incidence in both sexes. In the results for the combined variable of BMI and WC, women showed similar associations for general obesity measured by BMI and asthma regardless of abdominal obesity. However, an increased risk for asthma was observed in for overweight/obesity as measured by BMI only in men with abdominal obesity. Men without abdominal obesity did not show an increased risk of asthma even in obesity based on BMI.

**Table 3:** Subgroup analysis for asthma during four-year follow-up (2010–2013) according to body mass index and waist circumference by sex

		Men		Women	
		Case/N	HR (95% CI)	Case/N	HR (95% CI)
Body Mass Index (BMI)					
Underweight (< 18.5 kg/m^2^)		160/2706	1.18 (1.01–1.39)	129/2353	0.95 (0.79–1.13)
Normal (18.5–< 23 kg/m^2^)		2182/47659	–	2306/40929	–
Overweight (23–< 25 kg/m^2^)		1846/42599	0.98 (0.92–1.05)	1683/27415	1.08 (1.01–1.15)
Obesity (25–< 30 kg/m^2^)		2214/48073	1.07 (1.01–1.13)	1967/28287	1.21 (1.13–1.28)
Severe obesity (≥30 kg/m^2^)		155/2987	1.23 (1.05–1.45)	222/3011	1.28 (1.12–1.47)
Waist circumference (WC)					
Men	Women				
< 80 cm	< 75 cm	1451/33521	–	1796/33307	–
80–< 90 cm	75–< 85 cm	3435/77420	1.05 (0.99–1.12)	2985/46929	1.14 (1.08–1.21)
90–< 100 cm	85–< 95 cm	1483/29954	1.13 (1.05–1.21)	1314/18686	1.23 (1.14–1.32)
≥ 100 cm	≥ 95 cm	188/3129	1.34 (1.16–1.57)	212/3073	1.19 (1.03–1.37)
Combination of BMI & WC					
No abdominal obesity & Underweight/Normal (< 23 kg/m^2^)		2292/49523	–	2368/42074	–
No abdominal obesity & Overweight (23–< 25 kg/m^2^)		1548/37389	0.95 (0.89–1.01)	1427/23721	1.07 (1.00–1.14)
No abdominal obesity & Obesity (≥25 kg/m^2^)		1046/24029	1.04 (0.96–1.12)	986/14441	1.21 (1.12–1.30)
Abdominal obesity ^*^ & Underweight/Normal (< 23 kg/m^2^)		50/842	1.08 (0.82–1.43)	67/1208	0.90 (0.71–1.15)
Abdominal obesity ^*^ & Overweight (23–< 25 kg/m^2^)		298/5210	1.15 (1.02–1.29)	256/3694	1.15 (1.01–1.31)
Abdominal obesity ^*^ & Obesity (≥25 kg/m^2^)		1323/27031	1.09 (1.02–1.17)	1203/16857	1.22 (1.13–1.31)

Subgroup analysis was performed using BMI and waist circumference measured in 2008/2009 excluding subjects with asthma diagnosis before the follow-up period. Separate models were developed for BMI, WC and the combination of BMI and WC after adjusting for age, insurance type, household income, smoking, drinking and physical activity

^*^ Abdominal obesity was defined as waist circumference > 100 cm in men or 95 cm in women

Sensitivity analysis was conducted using two- and four-year lag times before the follow-up for asthma development. The association patterns between BMI and asthma were similar, although HRs were slightly decreased (Additional file 1: Table S1).

## Discussion

We defined pre-existing asthma based on the diagnosis of asthma with ICD-10 codes (J45 or J46) regardless of drug prescription in the selection process for the study sample, whereas newly diagnosed asthma was defined using both asthma diagnosis and drug prescription during the follow-up period for asthma incidence. A previous study by Dombkowski et al. explored the accuracy of several case definitions for asthma based on the claims database [41]. They compared the positive predictive values of different case definitions including participants taking one or more asthma medications (Rx cases) and those who were not taking asthma medications despite having an asthma diagnosis (Dx cases). They reported a higher accuracy for the definition of Rx cases than those of Dx cases. Thus, when we focus on the accurate detection of new asthma diagnoses, it may be more appropriate to consider both drug prescription and asthma diagnosis as a definition for asthma. However, it is more important to fully exclude patients with underlying asthma from the sample selection process. If there were patients who had not been prescribed drugs due to the temporary improvement of symptoms, pre-existing asthma cannot be detected using the same definition as for newly diagnosed asthma. Therefore, two different definitions of asthma were used to increase the diagnosis accuracy and exclude the possibility of underlying asthma prevalence.

Asthma incidence increased with age in both sexes and the increasing trend in terms of age was more prominent among men than among women. In addition, earlier studies revealed a higher asthma incidence among older individuals than among middle-aged individuals, particularly among men. For example, the asthma incidence in a Canadian population was 3.7 (2.8) and 4.2 (3.2) per 1000 person-years in individuals aged 40–69 and ≥ 70 years for 2000–2001 (2004–2005), respectively. The increment of the asthma incidence between middle-aged and elderly individuals was higher among men than among women (men, 40–69 years: 2.2 and ≥ 70 years: 3.1; women, 40–69 years: 3.3 and ≥ 70 years: 3.4; unit: per 1000 people) [44]. The rapid increase in asthma incidence among men might be related to sex hormones associated with the functions of epithelial cells. For example, progesterone inhibits the beat frequency of cilia and its receptor is expressed in the airway epithelium, which may affect mucociliary clearance during the menstruation cycle in women [45]. Indeed, asthma is more prevalent and severe after puberty in women [46, 47]. Likewise, menopausal women had a significantly lower risk for asthma than premenopausal women [48]. Therefore, our result that the incidence of asthma among older men was relatively higher than among older women might be explained by the effect of sex hormones in middle-aged and older individuals.

Although our results showed that the asthma incidence differed between men and women, the deleterious effects of obesity according to BMI on asthma were similar for both sexes. Several prospective studies have shown sex-specific associations between obesity in terms of BMI and asthma [18, 21, 22]. However, a meta-analysis of seven cohort studies with heterogeneous results concluded that obese individuals are almost at a twofold higher risk for asthma than those with normal weight in both sexes [49]. Although the strength of the obesity effect (BMI ≥30 kg/m^2^) on asthma was slightly lower (20–40% increased risk) compared to the results of previous studies (OR or RR 1.5–3.0) [16–25, 27, 50], a cautious comparison should be made because of the inconsistent reference category, different races (Western vs. Asian) and participants’ varying ages (mainly young to middle-aged adults vs. middle-aged and older adults). The findings in our study indicate that asthma is associated with obesity regardless of sex in middle-aged and older populations in Korea.

The underlying mechanism responsible for the association between obesity and asthma has not been fully elucidated. However, the following are several plausible relationships: *i*) systemic inflammation modulated by adipokines and adipose tissues, *ii*) mechanical changes in the respiratory system and *iii*) comorbidities. Adipose tissue secretes various pro- and anti-inflammatory adipokines that modulate inflammation [51]. An excess of pro-inflammatory adipokines (leptin, TNF-α, IL-6, etc.) may be responsible for the association between asthma and obesity. Pro-inflammatory molecules such as TNF-α and IL-6 inhibit the production of adiponectin and typical anti-inflammatory adipokines. The serum concentrations of pro-inflammatory adipokines increased with obesity, whereas serum adiponectin concentrations were lower in obese individuals [52, 53]. Thus, chronic systemic inflammation in obese individuals may increase their susceptibility to airway obstruction and bronchial hyper-responsiveness [54]. In addition, obesity causes adverse mechanical changes in lung volume and airway resistance due to increased intra-abdominal pressure, reduced diaphragm movement and changes in chest-wall properties [55]. Finally, common comorbidities such as obstructive sleep apnoea or gastroesophageal reflux disease could also affect the association between obesity and asthma [56].

We observed a sex difference in the association between underweight status and asthma incidence. In our results, underweight men based on BMI had a higher risk of asthma development than men in normal BMI ranges but the association was non-significant in women. Although the association between underweight status and asthma is still unclear, several empirical studies have shown a higher incidence of asthma in underweight subjects compared to the normal group [16, 19, 22, 27]. Moreover, underweight status has been associated with airway hyperresponsiveness (AHR), which is one of asthma’s most important pathophysiological features [35, 57]. For example, a study involving Korean asthma patients revealed a negative association between BMI and AHR [57]. Another study demonstrated an elevated risk of AHR in low BMI men during a four-year follow-up period [35]. These studies suggest that underweight status may affect the development of asthma through a different pathway from obesity. However, the underweight–asthma association remains to be defined in various populations with further studies, particularly regarding the sex difference.

It was found that the combined effect of abdominal obesity and BMI was also different between the sexes. Our results showed that only ‘obese men with abdominal obesity’ had a higher risk for asthma. ‘Obese men without abdominal obesity’ did not have an increased risk of asthma compared to those with ‘normal weight without abdominal obesity’. Meanwhile, the risk for asthma in women was elevated in general obesity based on BMI regardless of waist size. These results might be because VAT distribution showed age and sex differences. Abdominal obesity is characterised as increased adipose tissue surrounding the intra-abdominal organs, which is known as VAT [28]. The incremental amount of VAT according to WC was greater in men than in women [58, 59]. Furthermore, the slope of incremental VAT was steeper in older adults than in younger adults of either sex [59]. Therefore, the effect of abdominal obesity may be more prominent in older men.

Our study showed that health-related behaviours such as smoking, drinking and physical activity were associated with asthma risk. Similar to previous results [60, 61], smoking was a risk factor for asthma in our study because cigarette smoking can modify inflammation, which is associated with asthma [62]. Drinking was associated with a lower risk of asthma for women in our study, which is inconsistent with a previous study that found a U-shaped association with the risk for asthma [12]. This inconsistency in the association was attributed to differences in drinking behaviours, such as the preferred beverage type and number of consumed drinks, which can substantially change the association pattern. Our results showed that frequent exercise had a protective effect on asthma in men but the opposite effect in women. A previous meta-analysis also showed the inconsistency of the association between physical activity and asthma because of hidden effects including sex difference [10]. We only considered the frequency of physical activity, thus sex differences in the strength or duration of exercise could affect the results.

This study has several limitations. First, there was uncertainty regarding the accuracy of the definition of asthma using ICD-10 codes in the NHIS claims database. The differential diagnosis of chronic obstructive pulmonary disease (COPD) and asthma in primary care is challenging [63]. Thus, the asthma patients in our data could also have COPD. Although the positive predictive value of asthma in children was > 90% using the case definition of children prescribed one or more asthma medications in the claims database [41], we could not rule out the possibility that the number of patients with asthma may be either over- or underestimated. Second, we selected participants who had their BMI measured at the baseline and/or 2008–2009, which may have led to selection bias. However, the distributions of the baseline characteristics including obesity were similar to those in the original dataset and other nationally representative data such as the Korea National Health and Nutrition Examination Survey (data not shown) [64]. In addition, BMI in 2008–2009 was similar to the baseline BMI. Third, health-related behaviours such as smoking, drinking and physical activity were measured using self-reported questionnaires. The accuracy of the self-reported health behaviours has been explored previously and the results indicate the under-reporting of risky behaviours [65, 66]. This limitation could lead to an underestimation of the effect of health-related behaviours on asthma.

## Conclusions

This study showed an association between modifiable risk factors and asthma incidence in later life. Lifestyle factors including obesity, smoking, drinking and physical activity had independent effects on asthma incidence. Our results suggest that weight control and a healthy lifestyle can help prevent asthma.

## Supplementary information

**Additional file 1: Table S1.** Sensitivity analysis for association between baseline BMI (2002–2003) and asthma incidence according to various lag time by sex. This analysis was performed to confirm the similarity of the associations between the risk factors and asthma when using different start dates.

## Data Availability

The NHIS-HEALS data are distributed to registered user through the official website of NHIS data sharing service (https://nhiss.nhis.or.kr/bd/ay/bdaya001iv.do). After the evaluation of research proposal by NHIS review committee, registered user can receive special access privileges to the data.

## Abbreviations

AHR: Airway Hyperresponsiveness
BMI: Body Mass Index
CIs: Confidence Intervals
HRs: Hazard Ratios
ICD-10: International Classification of Disease, 10th Edition
NHIS-HEALS: National Health Insurance Service-Health Screening Cohort
VAT: Visceral Adipose Tissue
WC: Waist Circumference
