# Supplementary material for: Sex differences in the association between asthma incidence and modifiable risk factors in Korean middle-aged and older adults: NHIS-HEALS 10-year cohort
Source: BMC Pulm Med. 2019 Dec 16;19:248. doi: 10.1186/s12890-019-1023-3 (PMC6916451; doi:10.1186/s12890-019-1023-3)
Supplement: Supplementary file 1 — Additional file 1: Table S1. Sensitivity analysis for association between baseline BMI (2002–2003) and asthma incidence according to various lag time by sex. This analysis was performed to confirm the similarity of the associations between the risk factors and asthma when using different start dates. [file 12890_2019_1023_MOESM1_ESM.docx]

| Supplementary table 1. Sensitivity analysis for association between baseline BMI (2002-2003) and asthma incidence according to various lag time by sex. | | | | | | | |
| --- | --- | --- | --- | --- | --- | --- | --- |
|  | Men | | |  | Women | | |
|  | 8-year follow-up (2006-2013) | 6-year follow-up (2008-2013) | 4-year follow-up (2010-2013) |  | 8-year follow-up (2006-2013) | 6-year follow-up (2008-2013) | 4-year follow-up (2010-2013) |
|  | n= 248,479 | n=241,008 | n=232,880 |  | n=199,790 | n=193,562 | n=186,741 |
|  | HR (95% CI) | HR (95% CI) | HR (96% CI) |  | HR (95% CI) | HR (95% CI) | HR (96% CI) |
| Age |  |  |  |  |  |  |  |
| 40~49 | - | - | - |  | - | - | - |
| 50~59 | 1.54 (1.49-1.59) | 1.54 (1.48-1.59) | 1.54 (1.48-1.60) |  | 1.25 (1.21-1.29) | 1.24 (1.20-1.28) | 1.22 (1.18-1.27) |
| 60~69 | 2.37 (2.28-2.45) | 2.35 (2.26-2.44) | 2.24 (2.14-2.35) |  | 1.50 (1.45-1.54) | 1.46 (1.41-1.51) | 1.39 (1.34-1.45) |
| ≥70 | 3.50 (3.32-3.68) | 3.37 (3.18-3.57) | 3.19 (2.97-3.42) |  | 1.59 (1.51-1.67) | 1.54 (1.46-1.63) | 1.46 (1.36-1.56) |
| Insurance type |  |  |  |  |  |  |  |
| Self-employed insured | - | - | - |  | - | - | - |
| Employed insured | 0.90 (0.87-0.92) | 0.89 (0.87-0.92) | 0.89 (0.86-0.92) |  | 0.98 (0.95-1.00) | 0.98 (0.95-1.01) | 0.97 (0.94-1.00) |
| Medical aid beneficiary | 1.89 (1.34-2.66) | 1.86 (1.26-2.76) | 1.60 (0.96-2.66) |  | 1.37 (1.05-1.79) | 1.21 (0.88-1.67) | 0.86 (0.55-1.35) |
| Household income |  |  |  |  |  |  |  |
| Low | - | - | - |  | - | - | - |
| Med | 0.95 (0.91-0.98) | 0.93 (0.89-0.97) | 0.93 (0.88-0.98) |  | 0.98 (0.95-1.01) | 0.98 (0.94-1.02) | 0.97 (0.93-1.01) |
| High | 0.95 (0.91-0.99) | 0.94 (0.90-0.98) | 0.93 (0.88-0.98) |  | 0.96 (0.93-0.99) | 0.96 (0.92-0.99) | 0.93 (0.89-0.97) |
| Body Mass Index ^*^ |  |  |  |  |  |  |  |
| Underweight(<18.5 kg/m^2^) | 1.31 (1.21-1.42) | 1.22 (1.11-1.33) | 1.23 (1.10-1.36) |  | 0.99 (0.91-1.08) | 0.99 (0.90-1.09) | 0.94 (0.83-1.05) |
| Normal (18.5~<23 kg/m^2^) | - | - | - |  | - | - | - |
| Overweight (23~<25 kg/m^2^) | 0.99 (0.96-1.02) | 0.99 (0.96-1.03) | 1.00 (0.96-1.04) |  | 1.10 (1.07-1.14) | 1.08 (1.04-1.12) | 1.09 (1.04-1.13) |
| Obesity (25~<30 kg/m^2^) | 1.04 (1.01-1.07) | 1.03 (1.00-1.07) | 1.04 (0.99-1.08) |  | 1.22 (1.19-1.26) | 1.19 (1.16-1.23) | 1.20 (1.15-1.25) |
| Severe obesity (≥30 kg/m^2^) | 1.23 (1.12-1.34) | 1.20 (1.09-1.33) | 1.19 (1.06-1.33) |  | 1.36 (1.28-1.45) | 1.32 (1.23-1.41) | 1.31 (1.21-1.42) |
| Smoking status |  |  |  |  |  |  |  |
| Unknown | 1.06 (0.99-1.14) | 1.07 (0.99-1.16) | 1.05 (0.96-1.15) |  | 1.02 (0.93-1.12) | 1.02 (0.92-1.13) | 1.03 (0.92-1.16) |
| Never smoker | - | - | - |  | - | - | - |
| Past smoker | 1.11 (1.07-1.15) | 1.11 (1.06-1.16) | 1.10 (1.05-1.16) |  | 1.11 (0.98-1.26) | 1.04 (0.91-1.20) | 1.05 (0.89-1.24) |
| Current smoker | 1.08 (1.05-1.11) | 1.08 (1.04-1.11) | 1.08 (1.03-1.12) |  | 1.38 (1.29-1.48) | 1.32 (1.22-1.43) | 1.29 (1.18-1.42) |
| Drinking status |  |  |  |  |  |  |  |
| Unknown | 0.86 (0.76-0.98) | 0.87 (0.76-1.00) | 0.88 (0.75-1.03) |  | 0.98 (0.88-1.10) | 0.99 (0.88-1.12) | 0.98 (0.84-1.13) |
| None | - | - | - |  | - | - | - |
| <Twice a week | 0.87 (0.84-0.89) | 0.88 (0.85-0.91) | 0.87 (0.84-0.91) |  | 0.99 (0.96-1.03) | 0.99 (0.96-1.03) | 0.98 (0.94-1.03) |
| ≥Three times a week | 0.87 (0.84-0.90) | 0.87 (0.84-0.91) | 0.89 (0.84-0.93) |  | 1.05 (0.96-1.14) | 1.07 (0.97-1.18) | 1.09 (0.97-1.22) |
| Physical activity |  |  |  |  |  |  |  |
| Unknown | 1.08 (0.99-1.17) | 1.06 (0.97-1.17) | 1.07 (0.96-1.19) |  | 1.04 (0.96-1.13) | 1.05 (0.96-1.15) | 1.08 (0.98-1.20) |
| None | - | - | - |  | - | - | - |
| <Twice a week | 0.97 (0.93-1.00) | 0.97 (0.93-1.00) | 0.97 (0.93-1.01) |  | 1.01 (0.97-1.04) | 1.02 (0.98-1.06) | 1.03 (0.98-1.07) |
| ≥Three times a week | 0.97 (0.94-1.00) | 0.97 (0.93-1.00) | 0.98 (0.94-1.02) |  | 1.05 (1.01-1.08) | 1.05 (1.01-1.09) | 1.05 (1.00-1.10) |
| HR=hazard ratios; CI= confidence intervals  ^*^ BMI measurements in 2002/2003 | |  |  |  |  |  |  |
